# Crystal structure and substrate interactions of an unusual fungal non-CBM carrying GH26 endo-*β*-mannanase from *Yunnania penicillata*

**DOI:** 10.1038/s41598-019-38602-x

**Published:** 2019-02-19

**Authors:** Pernille von Freiesleben, Olga V. Moroz, Elena Blagova, Mathias Wiemann, Nikolaj Spodsberg, Jane W. Agger, Gideon J. Davies, Keith S. Wilson, Henrik Stålbrand, Anne S. Meyer, Kristian B. R. M. Krogh

**Affiliations:** 1 Novozymes A/S, Krogshøjvej 36, 2880 Bagsværd, Denmark; 2 DTU Bioengineering, Department of Biotechnology and Biomedicine, Building 221, Technical University of Denmark, 2800 Kgs. Lyngby, Denmark; 3 York Structural Biology Laboratory, Department of Chemistry, University of York, York, YO10 5DD UK; 4 Department of Biochemistry and Structural Biology, Center for Molecular Protein Science, Lund University, PO Box 124, SE-221 00 Lund, Sweden

## Abstract

Endo-*β*(1 → 4)-mannanases (endomannanases) catalyse degradation of *β*-mannans, an abundant class of plant polysaccharides. This study investigates structural features and substrate binding of *Ypen*Man26A, a non-CBM carrying endomannanase from *Yunnania penicillata*. Structural and sequence comparisons to other fungal family GH26 endomannanases showed high sequence similarities and conserved binding residues, indicating that fungal GH26 endomannanases accommodate galactopyranosyl units in the −3 and −2 subsites. Two striking amino acid differences in the active site were found when the *Ypen*Man26A structure was compared to a homology model of *Wsp*.Man26A from *Westerdykella sp*. and the sequences of nine other fungal GH26 endomannanases. Two *Ypen*Man26A mutants, W110H and D37T, inspired by differences observed in *Wsp*.Man26A, produced a shift in how mannopentaose bound across the active site cleft and a decreased affinity for galactose in the −2 subsite, respectively, compared to *Ypen*Man26A. *Ypen*Man26A was moreover found to have a flexible surface loop in the position where *Pans*Man26A from *Podospora anserina* has an *α*-helix (α9) which interacts with its family 35 CBM. Sequence alignment inferred that the core structure of fungal GH26 endomannanases differ depending on the natural presence of this type of CBM. These new findings have implications for selecting and optimising these enzymes for galactomannandegradation.

## Introduction

**Figure 1:**
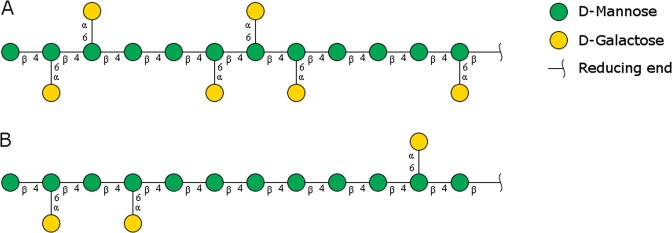
Schematic illustration of the two galactomannans (**A**) guar gum and (**B**) locust bean gum, with different degree and pattern of galactose substitutions on the *β*-mannan backbone^12^. Sugars shown using the Consortium for Functional Glycomics notation^59^. Both polymers continue towards the reducing end, having a degree of polymerization around 1500 for locust bean gum and 900 for guar gum^12^.

Endo-*β*(1 → 4)-mannanases (endomannanases, EC 3.2.1.78) are important enzymes, catalysing the degradation of abundant plant *β*-mannans (hereafter mannan) in nature. Endomannanases are currently used in various applications including plant biomass conversion^1^, food and feed^2,3^, detergent formulations^4^ and oil drilling^5^. An understanding of the intimate interactions between endomannanases and their substrates is key to optimising their utilisation and industrial performance. Mannan is an abundant type of hemicellulose in nature, primarily found in the secondary plant cell walls of softwood (coniferous trees). Mannans also serve as storage polysaccharides in certain seeds^6^. Mannans are composed of a linear backbone containing D-mannopyranosyl residues (linear mannans) or D-mannopyranosyl and D-glucopyranosyl residues organised in an alternating manner (glucomannans) linked by *β*-(1 → 4)-linkages. The backbone can be decorated with *α*-(1 → 6)-linked D-galactopyranosyl groups (galactomannans or galactoglucomannans) and acetyl groups^6–8^ (examples of galactomannans are shown in Fig. 1). In the secondary plant cell walls of softwood, acetylated galactoglucomannans comprise approximately 25% of the wood dry matter^9–11^. Guar gum, produced from the seeds of the guar plant (*Cyamopsis tetragonolobus*) and locust bean gum, produced from the seeds of the carob tree (*Ceretonia siliqua*) are significant sources of galactomannans. Guar gum contains more galactopyranosyl groups (Gal:Man, 1:2) than locust bean gum (Gal:Man, 1:4)^6^. In locust bean gum, the distribution of galactopyranosyl side-groups is irregular with a high proportion of unsubstituted blocks, whereas in guar gum, the galactopyranosyl groups are more ordered and found mainly in pairs and triplets with few non-substituted regions^12^ (Fig. 1).

Endomannanases are the main enzymes which catalyse depolymerisation of mannan. Endomannanases catalyse cleavage of the *β*-(1 → 4)-linkages in mannans to produce mannooligosaccharides which may be further processed by e.g. the exo-acting *β*-mannosidases and *α*-galactosidases. Soluble substrates are often accessible to all these enzymes, but attack on mannan by endomannanases may also occur on water-insoluble substrate matrices^1,2,13^. Endomannanases are classified into four glycoside hydrolase (GH) families: 5, 26, 113 and 134 based on sequence similarity^14^. Endomannanases from families 5, 26, and 113 belong to clan GH-A and share the (*β*/*α*)_8_-TIM barrel fold and catalytic machinery, and catalyse the cleavage of the *O*-glycosidic bonds in the mannan backbone with net retention of the anomeric configuration^15–17^. In contrast, the newly identified GH134 endomannanases have a lysozyme-like fold and catalyse the hydrolysis of the mannan backbone via an inverting mechanism^18^. Fungal endomannanases known to date are predominantly categorised in family GH5 with a few in family GH26. Several GH26 endomannanases from different organisms have been characterised (e.g. *Cfim*Man26A from *Cellulomonas fimi* (2BVY)^19^, *Cjap*Man26A (1J9Y)^20^ and *Cjap*Man26C (2VX6)^21^ from *Cellvibrio japonicus*, *Bova*Man26A (4ZXO) and *Bova*Man26B from *Bacteroides ovatus*^22^ and *Rspe*Man26A from a symbiotic protist of the termite *Recticulitermes speratus* (3WDR)^23^). Fewer studies have focused on the fungal GH26 enzymes and only one crystal structure is available, namely that of *Pans*Man26A from *Podospora anserina*, 3ZM8^24^, which carries a family 35 carbohydrate-binding module (CBM35). *Pans*Man26A and the GH26 endomannanase from *Aspergillus nidulans*, *Anid*Man26A, were shown to have a significant −4 subsite, and to accommodate galactopyranosyl units not only in the −1 subsite, but also in −2 and +1, in contrast to the GH5 counterparts from *A. nidulans Anid*Man5A and *Anid*Man5C^24–26^. Several fungal GH26 endomannanases were found to have higher initial rates on soluble galactomannans than the tested GH5 endomannanases, with the GH26 endomannanase from *Yunnania penicillata, Ypen*Man26A, having the highest initial hydrolysis rate, closely followed by *Anid*Man26A and the GH26 endomannanase from *Westerdykella sp*, *Wsp*.Man26A^1^. However, the tested fungal GH26 endomannanases discriminated differently between the soluble mannans^1^, exemplified by the *Ypen*Man26A and the *Wsp*.Man26A which both had high initial hydrolysis rates on locust bean gum, but different rates on more heavily substituted galactomannan. While *Ypen*Man26A also showed high hydrolysis rate on guar gum, *Wsp*.Man26A appeared more restricted by the extra galactose substitutions.

Most fungal GH26 endomannanases have a CBM35^24,26,27^; a CBM family known to include members that bind *β*-mannans, uronic acids, *β*-1,3-galactan or α-1,6-galactopyranosyl residues on carbohydrate polymers^28,29^. The binding site of CBM35s has been reported to be located in between the loops connecting the *β*-strands and not on the concave surface presented by the *β*-strands^28,29^.

In the present study, the Michaelis-Menten kinetic parameters for *Ypen*Man26A were determined, the crystal structure in complex with a galactomannooligosaccharide was solved, and the amino acids involved in substrate interactions identified. The structure of this unusual fungal wild-type enzyme with no CBM35 was compared to the known *Pans*Man26A structure harbouring a CBM35 and by sequence alignment to seven other fungal GH26 endomannanases. The roles of selected substrate binding amino acids were evaluated from two *Ypen*Man26A mutants, D37T and W110H. The mutations were inspired by the sequence of *Wsp*.Man26A, an endomannanase seemingly more restricted by galactose substitutions than *Ypen*Man26A.

## Results

*Y. penicillata* possesses at least one protein with endomannanase activity^1^ (GenBank sequence ID AYU65281). This enzyme, studied in the current paper, has a signal peptide and a GH26 catalytic domain, but no CBM, in contrast to most known fungal GH26 endomannanases which carries a CBM35^1,24,27^. A gene encoding the catalytic domain, named *Ypen*Man26A, was cloned and expressed in *Aspergillus oryzae*. Based on a sequence alignment with the sequence of *Pans*Man26A, the two catalytic residues (previously identified for GH26 enzymes^30,31^), Glu165 and Glu257 in *Ypen*Man26A were identified, with Glu257 being the nucleophile, performing the nucleophilic attack on an anomeric carbon in the mannan backbone, and Glu165 the acid/base, which serves as proton donor and later deprotonates the glycosyl acceptor in the first and second step of the retaining catalytic mechanism respectively^15,32^. This mechanism is characteristic for Clan GH-A glycosyl hydrolases, such as GH26 endomannanases^15^. The Michaelis-Menten kinetic parameters with locust bean gum and guar gum were determined for *Ypen*Man26A. Interestingly, the *k*_cat_ on guar gum (636 s^−1^) was found to be higher than that on locust bean gum (475 s^−1^). Previous studies reported a decrease in hydrolytic rate of endomannanases going from less to more substituted galactomannans, such as from locust bean gum to guar gum^19,22,33^. It is thought that the galactose substitutions cause steric hindrance, making the mannan backbone less accessible to the enzyme^6,34^. As expected, the *K*_*M*_ was also higher on guar gum (2.2 mg/ml) than on locust bean gum (0.6 mg/ml) and the *k*_cat_/*K*_M_ therefore lower on guar gum (289 ml/(mg·s)) than on locust bean gum (792 ml/(mg·s)). Motivated by the desire to see how this enzyme accommodates and interacts with the galactopyranosyl groups in galactomannan, we sought to determine the crystal structure of *Ypen*Man26A in complex with a galactomannooligosaccharide. A *Ypen*Man26A acid/base substituted variant, E165Q, was made using synthetic oligonucleotides and PCR, replacing the codon GAG at position 165 with CAG. The variant was synthesised and expressed in *Aspergillus oryzae. N*-Deglycosylation of the purified wild type and the E165Q *Ypen*Man26A mutant using Endoglycosidase H, resulted in a small shift (~5 kDa) in the apparent molecular mass on SDS-PAGE (Fig. S1). These results confirm that *Ypen*Man26A is *N*-glycosylated, in agreement with the GPMAW (Lighthouse data) prediction.

### Structure of *Ypen*Man26A

**Figure 2:**
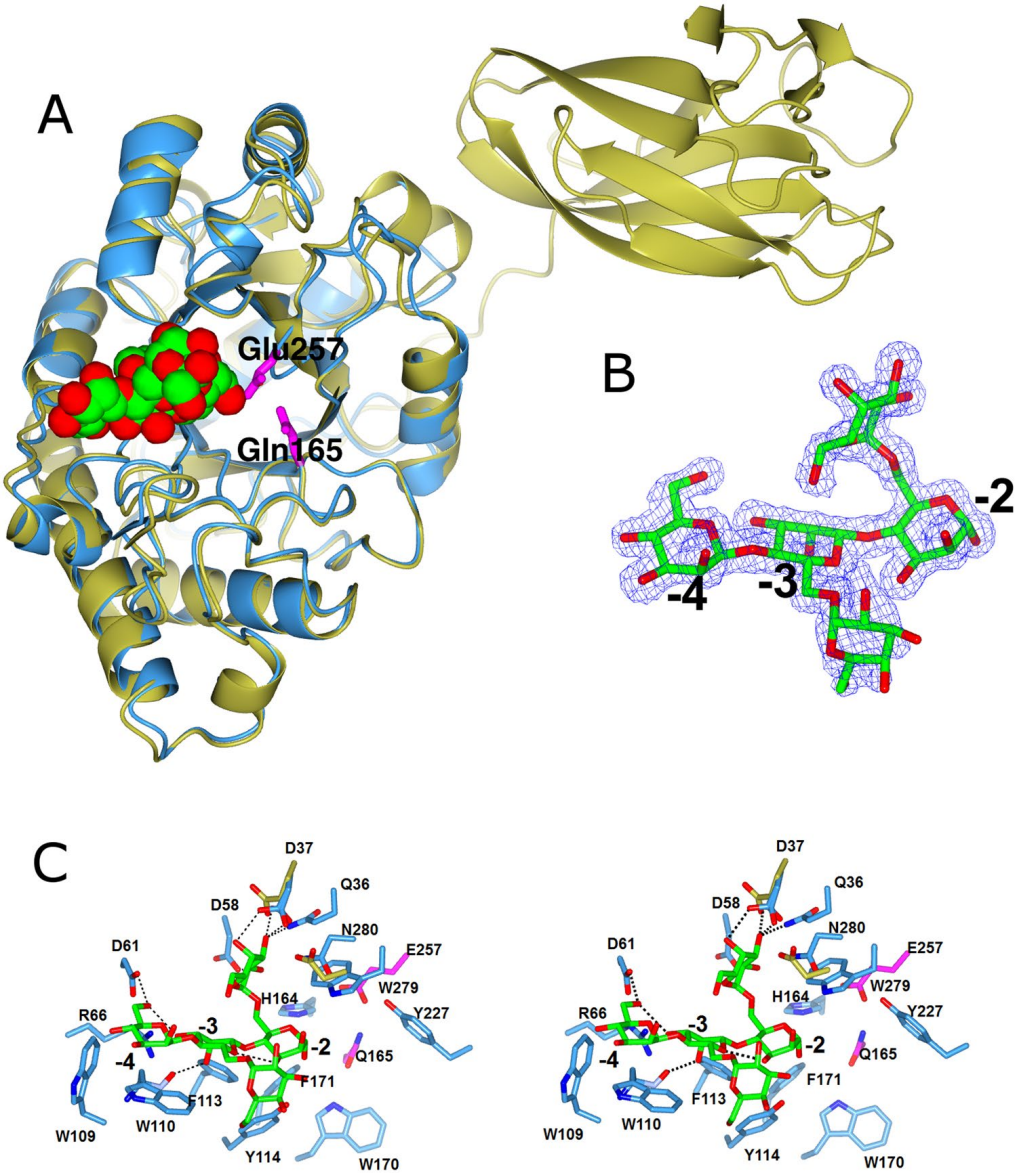
(**A**) The structure of *Ypen*Man26A (blue) superimposed with that of *Pans*Man26A (3ZM8^24^, gold). The *α*-6^2^-6^1^-di-galactosyl-mannotriose (MGG) ligand in *Ypen*Man26A (subsites −4 to −2) is shown as green cylinders and the active residues are shown in shades of pink (**B**) Observed electron density for MGG in the −4 to −2 subsites. The positive electron density REFMAC *F*_*o*_ − *F*_*c*_ map, contoured at 3.5 σ (0.37 e Å^−3^) is shown in blue, with phases calculated prior to the incorporation of any ligand atoms in refinement. (**C**) The organisation of binding subsites and the MGG ligand in the −4 to −2 subsites of *Ypen*Man26A (blue) compared with *Pans*Man26A (gold). *Pans*Man26A residues are only shown for those residues which differ from *Ypen*Man26A. All panels were drawn using *CCP*4*mg*^54^.

The structure of the deglycosylated *Ypen*Man26A acid/base substituted variant E165Q, in complex with a *α*-6^2^-6^1^-di-galactosyl-mannotriose (MGG), was solved by molecular replacement using the known structure of *Pans*Man26A^24^ as template, and refined at 1.36 Å resolution (Table 1). A *Ypen*Man26A E165A variant was also cloned but this variant was not successfully expressed. Neither the active *Ypen*Man26A nor the E165Q mutant crystallized as apoenzymes, suggesting that ligand binding resulted in increased stability and/or conformational changes leading to successful crystallogenesis. The *Ypen*Man26A chain can be traced from Ala1 to Val312 without breaks, and forms a (*β*/*α*)_8_-barrel fold (Fig. 2A) as expected. The active site was identified in the groove with the conserved catalytic residue Glu165 (acid/base) mutated to Gln, and the conserved catalytic residue Glu257 (nucleophile) (Fig. 2A)^30,31^, equivalent to those observed in *Pans*Man26A^24^. The low-activity *Ypen*Man26A E165Q variant showed an initial rate of hydrolysis of locust bean gum of 40 U/µmole enzyme (equivalent to a “turnover rate” of 0.7 s^−1^), which was roughly 360 fold lower than the rate exhibited by the wild type enzyme (15050 U/µmole enzyme, equivalent to a “turnover rate” of 251 s^−1^). 1 U was defined as the amount of endomannanase (in moles) required to release 1 µmole of reducing ends per minute, under the assay conditions specified in Methods. The low activity of the E165Q variant may be a consequence of the acid/base, and not the nucleophile, being substituted. An alternative explanation may be a consequence of the small risk (about 1/100) of translational misreading error or mis-incorporation of the wrong amino acid (as reported for *E. coli*)^35^ since the E165Q variant was made with only a single base change from codon GAG (Glu) to CAG (Gln). There is a single *N*-glycosylation site at Asn103, located on the external side of the barrel, with a residual *N*-acetylglucosamine (GlcNac). As expected, *Ypen*Man26A shows the highest structural similarity to other endomannanases (from both fungal, bacterial and protists origin) in family GH26 (Table 2). Judged from the Z-score (used by the DALI protein structure comparison server^36^ for ranking of structural matches) *Ypen*Man26A has the greatest structural similarity to *Pans*Man26A (3ZM8) followed by *Rspe*Man26C (3WRD) (Table 2).

**Table 1:** Data collection and refinement statistics of *Ypen*Man26A.

Data set^a^	MGG - *Yp*Man26 E165Q
PDB code	6HPF
*Data collection*	
Beamline	I04, Diamond, 2017.09.18
Space group	*P*6_5_22
Unit-cell parameters (Å)	*a* = 98.99, *b* = 98.99, *c* = 170.50
Resolution range (Å)	34.22–1.36 (1.38–1.36)
No. of reflections	1924268
Unique reflections	105928
Completeness (%)	100 (100)
CC_1/2_	1 (0.894)
Multiplicity	18.2 (18.5)
〈*I*/σ(*I*)〉	20.7(1.2)
R_merge_	0.058 (1.242)
R_r.i.m._^b^	0.061 (1.313)
*Refinement statistics*	
Percentage of R_free_ reflections	4.97
(%)R_cryst_ = Σ| |Fo| − |Fc| |/Σ|Fo| (%)	12.2
Free R factor (%)	14.4
Bond distances (Å)	0.017 (0.020)
Bond angles (°)	1.72 (1.92)
Chiral centres (Å^3^)	0.118 (0.200)
Planar groups (Å)	0.014 (0.021)
Average B value protein (Å^2^)	18
Average B value ligand (Å^2^)	24
Average B value water (Å^2^)	35
Molprobity score	0.81
Ramachandran favoured	97.4
Ramachandran outliers	0.37

^a^Values in parentheses correspond to the highest resolution shell. ^b^Estimated *R*_r.i.m._ = *R*_merge_ [*N*/(*N* − 1)]^1/2^, where *N* is the data multiplicity, and R_merge_ is defined as Σ |I − 〈I〉| /Σ I, where I is the intensity of the reflection. ^c^CC(1/2) values for Imean are calculated by splitting the data randomly in half. ^d^Ramachandran plot analysis was carried out using Molprobity^52^.

**Table 2:** The five closest structural matches to *Ypen*Man26A, calculated using the DALI protein structure comparison server^36^ (excluding duplicates).

Enzyme	PDB code	Z-score	R.m.s.d. (Å)	Sequence identity (%)	Residues aligned
PansMan26A, Podospora anserina^24^	3ZM8	48.1	1.0	46	309/444
*Rspe*Man26C, *Reticulitermes speratus*^23^	3WDR	42.0	1.5	36	298/330
*Bsub*Man26A, *Bacillus subtilis*^60^	2WHK	33.1	2.1	27	276/332
BCMan, *Bacillus subtilis*^61^	2QHA	33.1	2.1	27	276/336
*Bacillus subtilis*	3CBW	33.1	2.0	27	275/336

### Ligand binding to *Ypen*Man26A

Crystals of *Ypen*Man26A E165Q were obtained in the presence of *α*-6^4^-6^3^-di-galactosyl-mannopentaose (MGGMM) with the aim that the oligosaccharide would span the catalytic site. However, the electron density of the ligand was modelled as MGG situated in the −4 to −2 subsites (Fig. 2C). Since *Ypen*Man26A E165Q was not completely inactive, it is likely that the residual activity has caused hydrolysis of the MGGMM between the backbone monomers in the −1 and +1 subsites, after which MGG migrated to span the subsite −4 to −2, indicating high ligand affinity in these subsites. This is supported by the observation that wild type *Ypen*Man26A also produces MGG as a major hydrolysis product (discussed further below). The electron density of MGG is clear and unambiguous, except for the galactopyranosyl unit in the −3 subsite, which points out of the binding cleft (Fig. 2B). The B values for the galactopyranosyl residue in the −3 subsite are also higher (between 34–63 Å^2^ for the C atoms), than for the galactopyranosyl unit in the −2 subsite (between 17–30 for the C atoms) or for the mannopyranosyl moieties (between 14–28 for the C atoms). All the interactions between the enzyme and the ligand are clearly defined, except for the flexible galactopyranosyl unit. There is electron density present near the mutated Q165, which is remote from the ligand, and was described as acetate, which fits the density well. There was no acetate in the crystallisation buffer, but most probably it was a contaminant during purification or crystallisation, or was present in the cell growth media, similar to the unknown ligand described as propionate in 5G4Z^37^.

Like *Pans*Man26A, *Ypen*Man26A has eight large loops that form a deep cleft at the active centre and are involved in binding of the substrate: loop 1 (36–39), loop 2 (60–73), loop 3 (95–131), loop 4 (166–179), loop 5 (207–211), loop 6 (227–235), loop 7 (259–263), and loop 8(279–291). The −1 and +1 subsites of *Ypen*Man26A are similar to other fungal and bacterial GH26 endomannanases (e.g. *Pans*Man26A, *Cjap*Man26A, *Cfim*Man26A^19,20,24^) with the conserved residues His164, Trp170, Phe171, Tyr227, Trp279 (Fig. 2C). As described for the homologous enzymes^19,20,24^, *Ypen*Man26A Tyr227 is involved in a hydrogen bond with the catalytic nucleophile Glu257 whilst the aromatic amino acids Trp170 and Trp279 stabilise the mannopyranose rings at the −1 and +1 subsites, respectively (Fig. 2C). Like *Pans*Man26A, *Ypen*Man26A displays a prominent −4 subsite, with stacking interactions between the mannopyranose ring and two aromatic residues W109 and W110 and hydrogen bonds between Asp61, Arg66 and the mannopyrannose ring (Fig. 2C). The −3 subsite appears weaker bound as judged from the ligand enzyme interactions. In the −2 subsite the two aromatic residues, Phe113 and Tyr114, equivalent to Phe248 and Tyr249 in *Pans*Man26A, stabilise the interactions with the mannopyranose unit. Previously, enzyme interactions with a galactopyranosyl substituent attached to a mannopyranosyl unit within the −1 subsite of *Cjap*Man26C have been described^21^. Interestingly, because of the captured ligand in the present study, it is possible to identify interactions between the galactopyranose unit and the *Ypen*Man26A in the −2 subsite not previously described. Gln36, Asp37, and Asp58 are involved in hydrogen bonds with the galactose residue. Asp37 has a double conformation in the crystal structure, possibly because the amino acid conformation shifts upon ligand binding. *Pans*Man26A has a Glu172 instead of the Asp37 in *Ypen*Man26A, but otherwise the enzymes have essentially identical environments for interactions with the galactose residue. Out of the six closest structural matches (Table 2), only *Pans*Man26A (3ZM8) accommodates galactopyranosyl residues in the −2 subsite like *Ypen*Man26A. A surface view of *Ypen*Man26A and *Cjap*Man26C (2VX6) with their ligands superimposed (the MGG from *Ypen*Man26A and a bound *α*-6^3^-galactosyl-mannotetraose (MGMM) in the −2 to +2 subsite of *Cjap*Man26C) shows that the ligands overlap nicely. The data thus indicate accommodation of galactopyranosyl residues in the −3, −2 and −1 subsites of both enzymes (Fig. S2). These superimpositions show that *Cjap*Man26C does not accommodate the galactopyranosyl unit in the −2 subsite, where the moiety is pointing into the enzyme structure, whereas *Ypen*Man26A accommodates galactopyranosyl moieties in −3, −2 and −1 (Fig. S2). The data also show that *Ypen*Man26A has a more open active site than *Cjap*Man26C (Fig. S2).

### Design of two *Ypen*Man26A variants – inspired by *Wsp*.Man26A

**Figure 3:**
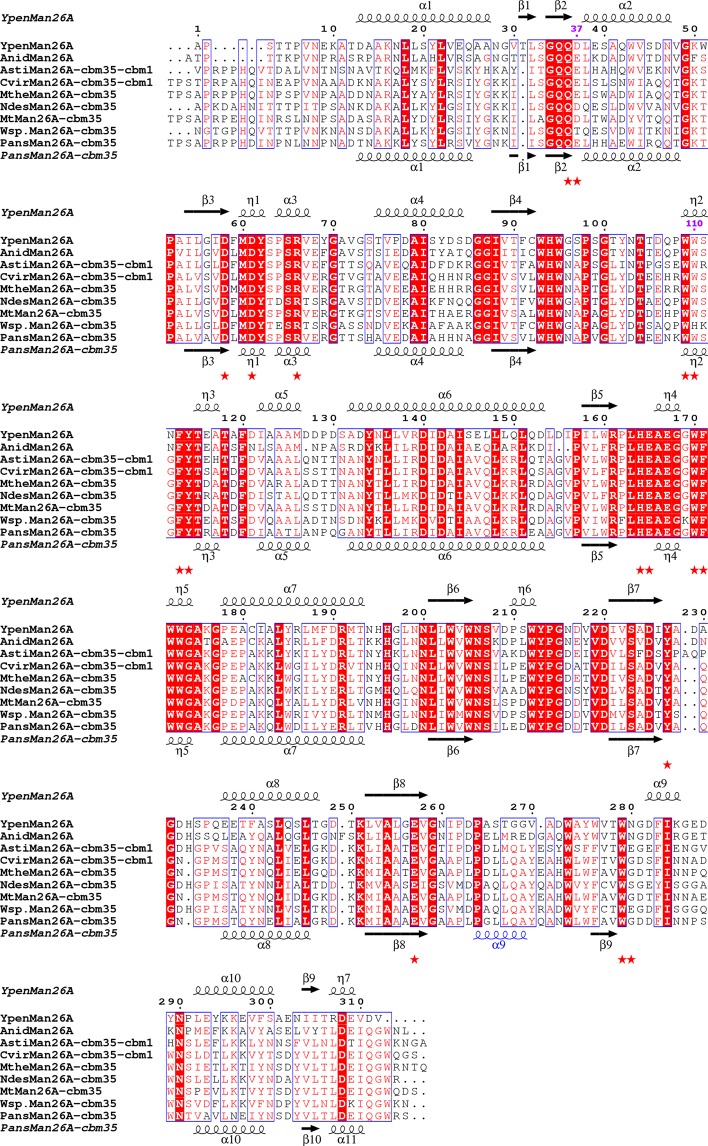
Sequence alignment of the catalytic GH26 core region from 9 fungal GH26 endomannanases. Secondary structure elements for *Ypen*Man26A and *Pans*Man26A are displayed above and below the alignment respectively. Mutated residues D37 and W110 (lilac) and residues involved in ligand binding (red stars) in the *Ypen*Man26A structure including the two catalytic residues. The *α*-helix in *Pans*Man26A (α9) which is nearest the CBM35 and which is a surface loop in *Ypen*Man26A is coloured blue. Identical residues are shown in white on red background. Highly similar residues (when the similarity score assigned to one column is above 0.7) are coloured red and framed in a blue box. The GH26 core sequence of *Ypen*Man26A (AYU65281), *Anid*Man26A (Q5AWB7), *Ascobolus stictoideus Asti*Man26A (BBW45412), *Collariella virescens Cvir*Man26A (BBW45415), *Mycothermus thermophiles Mthe*Man26A (MH208368), *Neoascochyta desmazieri Ndes*Man26A (MH208367), *Myceliophthora thermophila Mt*Man26A (99077), *Wsp*.Man26A (MH208369), *Pans*Man26A (B2AEP0) were aligned by MUSCLE^55^ and the figure was generated using ESPript 3 Web server^56^.

A sequence similarity search with the *Ypen*Man26A sequence, using the NCBI protein-protein BLAST (Basic Alignment Search Tool at http://www.ncbi.nlm.nih.gov/BLAST/, against the non-redundant protein sequences database)^38^, identified the *A. nidulans* GH26 endomannanase (Swissprot ID Q5AWB7^26^) with 67.5% amino acid identity as the closest characterised enzyme. A multiple sequence alignment of 9 fungal GH26 endomannanases showed that the amino acids that take part in ligand binding in *Ypen*Man26A are highly conserved (Fig. 3, red stars) (see later paragraph for discussion of differences between sequences of the GH26 core domains with and without a CBM35). However, *Wsp*.Man26A has two striking differences compared to *Ypen*Man26A and the other endomannanases. The first is in the −2 subsite (*Ypen*Man26A Asp37), where the analysed endomannanases have either an Asp or a Glu, while *Wsp*.Man26A has Thr (Fig. 3).

**Figure 4:**
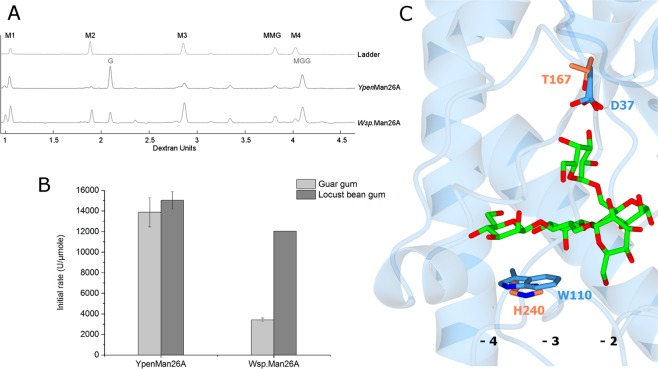
(**A**) Product profiles from guar gum hydrolysis by *Ypen*Man26A and *Wsp*.Man26A. Aligned electropherograms of product profiles at 30% guar gum conversion (max conversion). Migration of oligosaccharides is given in dextran units (DE). A ladder was run containing: mannose (M1, 0.9 DE), mannobiose (M2, 1.87 DE), mannotriose (M3, 2.85 DE), and α-6^1^-galactosyl-mannotriose (MMG, 3.81 DE). Migration of α-galactosyl-mannose (G, 2.10 DE), and α-6^2^-6^1^-di-galactosyl-mannotriose (MGG, 4.10 DE) was determined by von Freiesleben *et al*.^26^. (**B**) Initial reaction rates (U/µmole) by *Ypen*Man26A and *Wsp*.Man26A on galactomannans. Data are from von Freiesleben *et al*.^26^. Hydrolyses were carried out at 37 °C, pH 5 on guar gum (light grey) and locust bean gum (dark grey). Values are given as mean values ± SD (n = 2). (**C**) The structure of *Ypen*Man26A with MGG in the −4 to −2 subsites. The two differences in ligand binding amino acids between *Ypen*Man26A and a superimposed homology model of *Wsp*.Man26A are highlighted in blue and orange, respectively.

The second is in the −4 subsite (*Ypen*Man26A Trp110), where the tested endomannanases have Trp or Tyr, while *Wsp*.Man26A has His (Fig. 3). von Freiesleben *et al*.^1^ showed that *Ypen*Man26A and *Wsp*.Man26A differ in their substrate preferences for locust bean gum and guar gum. *Ypen*Man26A barely discriminated between the two substrates, whilst *Wsp*.Man26A had approximately four times higher initial hydrolysis rate on locust bean gum than on guar gum (Fig. 4B, data adapted from von Freiesleben *et al*.^1^), indicating that this enzyme was more hindered or had less affinity for the increased amount of galactose substitutions in guar gum. In the present study, the hydrolysis product profiles from full conversion of guar gum were analysed using the DNA sequencer-Assisted Saccharide analysis in High throughput (DASH) method^26,39^ (Fig. 4A).

*Ypen*Man26A produced primarily α-galactosyl-mannose (G, 2.10 DE) and α-6^2^-6^1^-di-galactosyl-mannotriose (MGG, 4.10 DE), whereas *Wsp*.Man26A in addition produced M2 and M3. To investigate if the difference in ligand interacting amino acids between *Ypen*Man26A and *Wsp*.Man26A (Fig. 4C) played a role in the observed differences in substrate preference and binding mode, two *Ypen*Man26A mutants, *Ypen*Man26A D37T and *Ypen*Man26A W110H, were designed, expressed and purified to electrophoretic purity (Table 3 and Fig. S3).

**Table 3:** The wild-type *Ypen*Man26A and the investigated variants.

Enzyme	Domains	Mw^a^ (kDa)	*Tm*^b^ (°C)
*Ypen*Man26A (GenBank sequence ID: AYU65281)	GH26	34.5	50
*Ypen*Man26A D37T	GH26	34.5	50
*Ypen*Man26A W110H	GH26	34.4	47

^a^Theoretical. ^b^The Thermal midpoint (*Tm*) at pH 5.

### Kinetics with galactomannans and MGGMM

The Michaelis-Menten kinetic parameters with locust bean gum and guar gum were determined for the two *Ypen*Man26A mutants D37T and W110H and compared with those reported for the wild type enzymes *Ypen*Man26A and *Wsp*.Man26A (Table 4). The wild-type *Ypen*Man26A had the highest *k*_cat_/*K*_M_ on both substrates, closely followed by the *Wsp*.Man26A on locust bean gum. The kinetic data for the two wild-type enzymes show that *Wsp*.Man26A is more compromised on the heavily substituted guar gum than *Ypen*Man26A; these results corroborate our previous data^1^. The wild type *Ypen*Man26A and the variant D37T had identical *K*_M_ values on locust bean gum, but D37T had a higher *K*_M_ than the wild type enzyme on guar gum. This result indicates that the D37T mutant has lower affinity for the galactose residues in the highly substituted guar gum than the wild type enzyme has. The reason that no difference in *K*_M_ values was observed on locust bean gum as substrate might be due to the presence of unsubstituted blocks of mannan in the locust bean gum mannan^12^. It is likely that both the wild type and the D37T variant catalyse the degradation of the unsubstituted, more easily accessible, part of the substrate first, so the initial rate reflects the enzyme affinity for the unsubstituted regions of the substrate. Guar gum is known to have no (or few) blocks without substitutions^12^. Based on the *K*_M_ value, the *Ypen*Man26A W110H variant appeared to have very low affinity for locust bean gum, when compared to the other enzymes. On guar gum galactomannan it was not possible to determine the kinetic parameters separately, because saturation was not reached, but the low *k*_cat_/*K*_M_ indicates low affinity or low hydrolysis rate.

**Table 4:** Kinetic parameters on locust bean gum and guar gum of the wild-type enzymes *Ypen*Man26A and *Wsp*.Man26A and the variants *Ypen*Man26A D37T and *Ypen*Man26A W110H.

Enzyme	Locust bean gum			Guar gum		
	*k*_cat_ (s^−1^)	*K*_M_ (mg/ml)	*k*_cat_/*K*_M_ (ml/(mg·s))	*k*_cat_ (s^−1^)	*K*_M_ (mg/ml)	*k*_cat_/*K*_M_ (ml/(mg·s))
*Ypen*Man26A	475 ± 5	0.6 ± 0.03	792 ± 40	636 ± 19	2.2 ± 0.2	289 ± 28
D37T	334 ± 6	0.6 ± 0.05	557 ± 47	473 ± 12	2.7 ± 0.2	175 ± 14
W110H	404 ± 18	10 ± 0.8	40 ± 4	n.d^**a**^	n.d^**a**^	17 ± 0.6
*Wsp*.Man26A	564 ± 26	0.8 ± 0.2	705 ± 179	271 ± 31	3.6 ± 1	75 ± 23

^a^Not determined (n.d), because saturation was not reached. Linear regression was used to determine *k*_cat_/*K*_M_ from the initial part of the Michaelis-Menten curve.

In addition, for the *Wsp*.Man26A substrate saturation was not fully reached, especially not on guar gum, resulting in relatively high standard deviation. *R*^2^ values for the fitted Michaelis-Menten curve for *Wsp*.Man26A were 0.90 and 0.91 on locust bean gum and guar gum, respectively.

**Figure 5:**
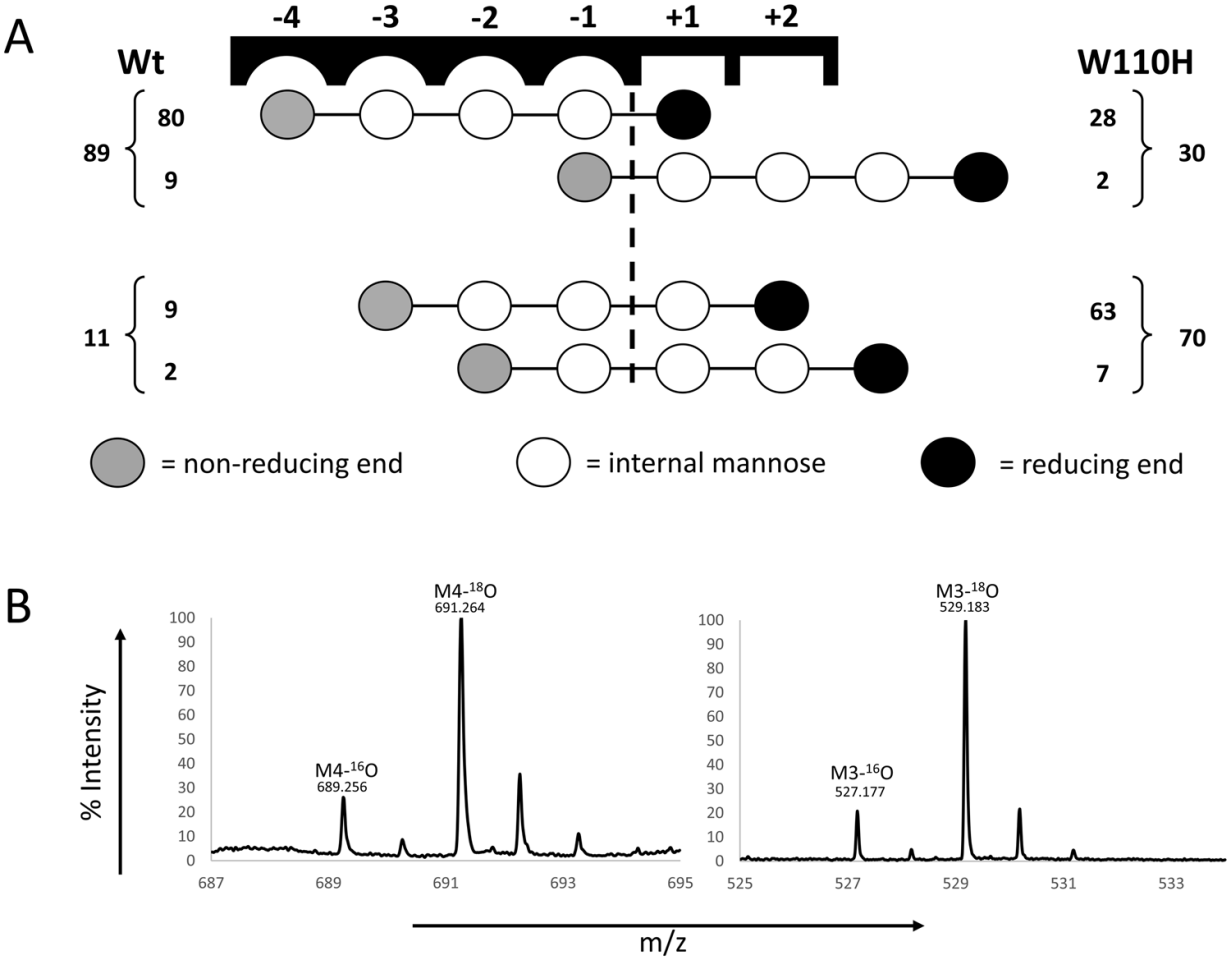
(**A**) Relative frequency of the productive binding modes of M5 for the *Ypen*Man26A wild-type and the W110H variant. Each circle represents a mannose unit. The dashed line between subsite −1 and +1 represents hydrolytic cleavage. The outmost numbers on respective side represent the total percentage of produced product, i.e. M4 and M1 or M3 and M2, determined by HPAEC-PAD quantification. These numbers were then combined with the individual ratios of labelled (^18^O) to unlabelled (^16^O) products (M4- and M3-species, respectively) (see panel B) to calculate the inner numbers which represent the relative frequency of each productive binding mode for the two enzymes. (**B**) Mass spectrometry peaks showing the major labelled (^18^O) hydrolysis product for *Ypen*Man26A wild-type (left) and W110H (right) together with unlabelled (^16^O) species of the same DP (M4 and M3 for the wild-type and W110H, respectively). From these spectra, a M4/M4-^18^O ratio of 1:8.9 and a M3/M3-^18^O ratio of 1:9.2 was calculated. The theoretical mass for M3 with a sodium adduct is 527.159 and the theoretical mass for M4 with a sodium adduct is 689.212.

To validate that the increase of *K*_M_ for *Ypen*Man26A D37T on the highly substituted guar gum galactomannan was caused by the change in the −2 subsite, *k*_cat_/*K*_M_ on MGGMM for the *Ypen*Man26A wild-type and the D37T mutant were determined by following substrate depletion at low substrate concentration (0.1 mM) by MS (Table 5). A novel MS based method with an internal standard was developed to allow these measurements (relevant spectra, extracted ion chromatograms and a standard curve are shown in Fig. S4). The reaction rate of MGGMM depletion could be described by the equation described by Matsui *et al*.^40^ (Fig. S5), which was used to determine *k*_cat_/*K*_M_. It is likely that MGGMM binds from the −4 to the +1 subsite in *Ypen*Man26A, and therefore accommodates the galactopyranosyl residues in the −3 and −2 subsite, as in the X-ray structure (Fig. 2C). This can be assumed because of the dominant M5 productive binding mode for *Ypen*Man26A from subsite −4 to +1 (see next section, Fig. 5) and the demonstrated capability of *Ypen*Man26A to accommodate the galactopyranosyl moiety in the −3 and −2 subsites (Fig. 2). Furthermore, *Anid*Man26A, which is the closest homologue to *Ypen*Man26A, was found to produce MGGM and M from MGGMM^26^.

**Table 5:** Kinetic efficiency on MGGMM for *Ypen*Man26A wild type and the variant *Ypen*Man26A D37T.

Enzyme	*k*_cat_/*K*_M_ (s^−1^·mM^−1^) on MGGMM
Wild type	84 ± 5
D37T	19 ± 2

The D37T variant had four times lower *k*_cat_/*K*_M_ on MGGMM than the wild type enzyme (84 vs 19 s^−1^·mM^−1^, Table 5), showing that the mutant has lower *k*_cat_ and /or higher *K*_M_ (probably a combination of both as for the individual kinetic parameters determined on guar gum). The observed *k*_cat_/*K*_M_ for the wild-type *Ypen*Man26A and the D37T variant is at the same level as *k*_cat_/*K*_M_’s reported for other fungal endomannanases on M5, which were found to range from 23–163 s^−1^·mM^−1^ for the GH5 endomannanases from *A. nidulans* and *Trichoderma reesei*^41^ and to be 22 s^−1^·mM^−1^ for *Pans*Man26A^24^. The bacterial GH26 endomannanase from *B. ovatus, Bova*Man26A, had a *k*_cat_/*K*_M_ of 247 s^−1^·mM^−1^ on M5^22^. This result emphasises that substitution of Asp37 with Thr decreases the affinity for the galactopyranosyl moiety in the −2 subsite. The lower *k*_cat_/*K*_M_ on MGGMM obtained for the D37T mutant compared to the wild type is consistent with the expected increase in distance between the galactopyranosyl unit and the amino acid residue when Asp is substituted with Thr (Fig. 4C).

### Productive binding of M5

M5 hydrolysis product analysis using HPAEC combined with solvent isotope labelling and mass spectrometry (MS) analysis^24,42^ was used to estimate the relative frequency of productive binding modes for the *Ypen*Man26A wild type and W110H mutant. The HPAEC product quantification showed a clear difference between the wild type and the W110H variant (Fig. S6), with the wild type preferring producing M4 and M1 (89% relative productive binding frequency) with little formation of M3 and M2 (11%). For the W110H mutant the major hydrolysis products were M3 and M2 (70%) as well as some M4 and M1 (30%). Because two productive binding modes can give rise to the same products (M5 can for example be hydrolysed into M4 and M1 through removal of the reducing end or the non-reducing end mannopyranosyl unit), the HPAEC data were combined with an *in situ* labelling, matrix-assisted laser desorption ionization time-of-flight mass spectrometry (MALDI-TOF MS) analysis procedure^24,42^ where M5 hydrolysis is performed in ^18^O-water, to obtain product ratios of ^18^O-labelled versus ordinary ^16^O-products. The newly formed reducing end will be labelled with ^18^O (heavy product) while the “leaving group” saccharide (light) of each catalytic event will not. With the MS analysis, it is thus possible to distinguish between M4 produced by M5 binding from subsite −4 to +1 (generating heavy M4) and M5 binding from subsite −1 to +4 (generating light M4). The heavy versus light product ratios obtained for M3 and M4 were used to calculate the relative binding frequencies of binding modes that generate these products, respectively (Fig. 5).

The data show that for wild-type *Ypen*Man26A, the dominant productive M5 binding mode is from subsite −4 to +1 (80% binding frequency) (Fig. 5), but that this mode is significantly reduced (28%) for the W110H mutant. Instead the dominant productive M5 binding mode is shifted to cover subsites −3 to +2 (63% binding frequency). This is probably a consequence of Trp110 in the −4 subsite being changed to His, resulting in a weaker subsite. It is also possible that the W110H substitution has caused a slight change in the global active site fold, resulting in slightly reduced thermostability (Table 3) and decreased activity (Table 4). However, on locust bean gum it is mainly *K*_M_ and to a smaller extend *k*_cat_ that changes when comparing the W110H variant with the *Ypen*Man26A wild-type, indicating that the affinity for the substrate is dramatically changed while the hydrolysis rate is affected to a lesser extent. These results suggest that the W110H substitution has caused changed to the binding subsites and not the overall fold.

### Differences in the catalytic GH26 domain in fungal endomannanases with and without CBM35

Most regions are highly conserved between *Ypen*Man26A and *Pans*Man26A (Fig. 2A,C), but *Ypen*Man26A lacks a N-terminal CBM35 domain. From the superimposition of the two crystal structures (Fig. 2A), it is seen that the main difference in the secondary structure between the core modules of the two enzymes is in the area which approaches the CBM35 of *Pans*Man26A, where *Pans*Man26A has an *α*-helix and *Ypen*Man26A a surface loop. Interactions occur through water between the Ala402 and Gln404 in the *Pans*Man26A core domain and the Leu58 and the Ser130 in its CBM35 and linker respectively. Couturier *et al*.^24^ also report that a hydrophobic patch comprising Leu58 and Leu130 on the surface of the CBM35 stands in front of a cluster of hydrophobic residues, Ala402, Tyr403 and Leu399 of the core domain^24^. These interactions would not be established if the *Pans*CBM35 were appended to the *Ypen*Man26A, because of differences in the amino acid sequence and the flexible nature of the surface loop. The multiple sequence alignment (Fig. 3) of the GH26 core domains of nine fungal GH26 endomannanases (two wild-type core enzymes, five with a N-terminal CBM35 and two with a CBM35 and a C-terminal CBM1), confirms variation in the region in and around α9 in *Pans*Man26A (Fig. 3, marked blue), the area of the core domain approaching the CBM35. The seven enzymes with a CBM35 have identical sequences to *Pans*Man26A (LQAY, for *Asti*Man26A it is MQLY), which forms an *α*-helix in *Pans*Man26A, while the two enzymes with no CBM35, have a different and seemingly more variable sequence (TGGV for *Ypen*Man26A and MRED for *Anid*Man26A). From this analysis, it seems that co-evolution has occurred between the GH26 core domain and the CBM35. It is likely that the core domain evolved to accommodate and maybe help position the CBM35. When the CBM35 is absent, *α*9 is not needed.

## Discussion

Data presented here add to the current understanding of fungal GH26 endomannanases, which appear to be conserved in their known functional characteristics. Characterised fungal GH26 endomannanases, including *Ypen*Man26A, have a characteristic ligand binding site with a strong – 4 subsite, and a dominant M5 binding mode from the −4 to +1 subsite^24,26,27^, in contrast to at least some fungal GH5 endomannanases (including *Pans*Man5A) which mainly bind M5 from the −3 to the +2 subsite^24^. To date, the fungal GH26 endomannanases which have been analysed with a focus on the accommodation of galactopyranosyl units, are able to degrade highly substituted galactomannans by allowing accommodation of galactose substitutions at least in the −3, −2, −1 and +1 subsites as judged by biochemical data and crystal structures. The biochemical data include the observations that *Pans*Man26A and *Anid*Man26A produce α-galactosylmannose (G) as their dominant hydrolysis product from guar gum galactomannan and *Anid*Man26A catalyses the hydrolysis of MGGMM to MGGM and mannose^26^. The structural data include the crystal structure of *Pans*Man26A^24^ and the homology model of *Anid*Man26A that both show an open active site cleft with space for galactose substitutions^26^. Furthermore, our current crystal structure of *Ypen*Man26A with bound MGG from the −4 to the −2 subsites and the observation that the amino acids participating in MGG binding in *Ypen*Man26A are highly conserved between studied GH26 endomannanases (Fig. 2), further support this hypothesis. Some fungal GH5 endomannanases, e.g. the *Tres*Man5A from *T. reesei*, have been found to accommodate galactopyranosyl residues in the −1 subsite^43^, but not in the −2 and +1 subsites^26^. Among the bacterial GH26 endomannanases there is a variation in their ability to accommodate multiple galactopyranosyl residues in the active site cleft, exemplified by *Bova*Man26A and *Bova*Man26B from *Bacteroides ovatus*^22^.

We show that a single mutation in the substrate binding amino acids can result in altered binding modes or substrate affinity as seen for the *Ypen*Man26 wild-type and mutants investigated in the present study. Of the 17 amino acids involved in ligand binding (including the two catalytic residues) only three residues were not conserved among the nine fungal GH26 endomannanases compared in this study (Fig. 3). In two of these changes *Wsp*.Man26A differed from the rest of the endomannanases. Mutation studies showed that W110H shifted the dominant productive M5 binding mode of *Ypen*Man26A from covering the −4 to +1 subsites to the −3 to +2 subsites, emphasising the importance of Trp110 in the strong −4 subsite. The D37T mutation lowered the affinity for a galactopyranosyl unit in the −2 subsite of *Ypen*Man26A. A third variation in ligand binding amino acids among the studied GH26 endomannanases was position Asn280 in *Ypen*Man26A (Fig. 3). This residue is not conserved between the nine fungal GH26 endomannanases, which might indicate that this residue is not important for ligand binding or it could contribute to different affinity for galactose in the −2 subsite, similar to the D37T mutation investigated in the present study. Indeed fungal GH26 endomannanases were shown to have different ratios between their initial rate on locust bean gum and on guar gum^1^, indicating variations in galactose affinity and/or tolerance, which perhaps can be explained by variations at this position (Asn280 in *Ypen*Man26A, Fig. 3). Detailed knowledge about binding mode and affinity for substitutions in different subsites is important when using these enzymes to produce specific oligosaccharides e.g. for prebiotics or alkyl mannooligosides.

As seen from the superimposition of *Ypen*Man26A and *Pans*Man26A (Fig. 2A) and the sequence alignment of nine fungal GH26 endomannanases (Fig. 3), the main difference in their catalytic domains appears to be in the area approaching the CBM35 (if present). The GH26 core module of the enzymes with a CBM35 seems to have evolved to harbour this big binding domain (15 kDa) in close proximity to the core, by aid of an α-helix (α9) whereas the wild-type enzymes with no CBM35, *Ypen*Man26A and *Anid*Man26A, have a less structured surface loop in this area. The α9-helix in *Pans*Man26A is situated with the end of the helix pointing directly into the site where the linker is attached to the CBM35. It is possible that this α-helix plays an important role in positioning of the CBM35. It is also possible that the position we see in the crystal structure of *Pans*Man26A is not that of the CBM35 in solution, and it is likely that the core domain and the CBM35 can come in even closer contact, perhaps facilitated by ligand binding. A similar event has been reported for processive GH9 endoglucanases, for which a CBM3c module were shown to align with the catalytic cleft of the GH9 module, presumably forming one functional entity^44^. The linker in these GH9 cellulases is wrapped around the core domain, similar to the linker in *Pans*Man26A^24^, and contributes significantly to the positioning of the CBM3c.

## Conclusions

This study identified important amino acids for binding galactomannan in the −4 to −2 subsites of *Ypen*Man26A, by solving and analysing its crystal structure in complex with MGG. Particularly the −2 subsite has multiple interactions with the galactopyranosyl side group. The study also highlights the high sequence similarity of known fungal GH26 endomannanases, with conserved ligand binding amino acids in the active site cleft. These results strongly indicate that the capability of accommodating multiple galactopyranosyl side-groups in the binding cleft is conserved among the fungal enzymes in the GH26 family. The two *Ypen*Man26A variants, W110H and D37T, showed that these changes shifted the dominant M5 binding mode from covering the −4 to +1 subsite to cover the −3 to +2 subsite and lowered the affinity for galactopyranosyl residues in the −2 subsite. The crystal structure of *Ypen*Man26A has a unique surface loop when compared to the crystal structure of *Pans*Man26A, which appears to be a consequence of the enzyme lacking a CBM35. Known fungal GH26 endomannanases, including *Ypen*Man26A, seem tailored for hydrolysing highly substituted galactomannans. Understanding the intimate enzyme-substrate interactions and the possibilities of changing product profiles and substrate affinities are important for fine-tuned optimization and utilization of these enzymes in industrial applications.

## Methods

### Materials

Locust bean gum (low viscosity; sodium borohydride reduced), guar gum (medium viscosity), mannobiose (M2), mannotriose (M3), mannotetraose (M4), mannopentaose (M5), α-6^1^-galactosyl-mannotriose (MMG), α-6^4^-6^3^-di-galactosyl-mannopentaose (MGGMM), and *α*-6^2^-6^3^-6^4^-tri-xylosyl-glucotetraose (XXXG) were purchased from Megazyme (Ireland). All other chemicals were purchased from Sigma (Germany), unless otherwise stated. Mobility markers, dextran ladder, and the DASHboard software for DASH analyses were kindly donated by Prof. Paul Dupree (University of Cambridge, UK).

### Construction of variants

The gene sequence encoding *Ypen*Man26A (GenBank sequence ID: AYU65281) was used to make the mutated constructs. E165Q was introduced into the gene sequence by PCR using synthetic oligonucleotides replacing the codon GAG position 165 of the mature peptide with CAG. PCR was conducted for the 5′ fragment and 3′ fragment separately using Phusion High-Fidelity DNA Polymerase (ThermoFisher Scientific) under the following conditions: 98 °C 2 min, 35 cycles at 98 °C for 10 sec, 72 °C for 150 sec, followed by 72 °C for 10 min. The PCR products were gel purified and used as template for a second round of PCR, using the gene flanking primers to amplify the full-length gene with the native signal peptide. The full-length PCR product was cloned into pDAu222^45^, an *Aspergillus* expression vector under the control of a NA2-tpi double promoter using the BamHI and XhoI restriction sites, and its sequence was determined. The resulting pDAu222-*Ypen*Man26A-E165Q expression vector was transformed into *A. oryzae* MT3568. MT3568 is an amdS (acetamidase) disrupted derivative of *A. oryzae* Jal_355^46^ in which pyrG auxotrophy was restored in the process of inactivating the *A. oryzae* amdS gene. Secretion of *Ypen*Man26A E165Q in the culture supernatant of the recombinant MT3568 clones was confirmed by SDS-PAGE.

Mutants containing the D37T and W110H substitutions respectively were made as synthetic full-length cDNA constructs with the native signal peptide (ThermoFisher Scientific) cloned into pDAu222 using the BamHI and XhoI restriction sites. For D37T the codon GAC of position 37 of the mature peptide was replaced with ACC. For W110H the codon TGG of position 110 of the mature peptide was replaced with CAC. The constructs were verified by sequencing and the resulting pDAu222 expression vectors were transformed into *A. oryzae* MT3568. Secretion of mutants in the culture supernatant of recombinant MT3568 clones was confirmed by SDS-PAGE.

### Expression and purification

The fungal wild-type GH26 endomannanases *Wsp*.Man26A and *Ypen*Man26A, as well as the *Ypen*Man26A mutants D37T, W110H and E165Q were recombinantly expressed in *A. oryzae* MT3568 an amdS^46^. The enzymes, wild-types and variants, were purified to electrophoretic purity using hydrophobic interaction and ion exchange chromatography. The inactive *Ypen*Man26A E165Q variant, used for crystallisation, was further purified using size-exclusion chromatography and deglycosylated with Endoglycosidase H (Roche). The identity of the purified endomannanases was validated with mass spectrometry analysing a tryptic digest of the protein band excised from a SDS-PAGE gel. Protein concentrations were determined by UV absorption at 280 nm using theoretical extinction coefficients (ε). ε at 280 nm of all proteins were estimated by GPMAW 9.20 (Lighthouse Data) and were based on mature proteins without modifications.

### Crystallisation

The inactive *Ypen*Man26A mutant E165Q was concentrated to 48 mg/ml, in 20 mM MES, 125 mM NaCl, pH 6 and aliquoted into 50 µl samples. Aliquots not used for immediate crystallisation trials were flash-frozen in liquid nitrogen and stored at −80 °C. Initial crystallisation screening was carried out using sitting-drop vapour-diffusion with drops set up using a *Mosquito Crystal* liquid handling robot (TTP LabTech, UK) with 150 nl protein solution plus 150 nl reservoir solution in 96-well format plates (MRC 2-well crystallisation microplate, Swissci, Switzerland) equilibrated against 54 µl reservoir solution. Experiments were carried out at room temperature with several commercial screens, for the protein on its own and in the presence of 5 mM MGGMM. The best hits were obtained in the AmSO_4_ suite (QIAGEN), for the ligand complex. The conditions were manually optimised in a 24-well Linbro dish, in hanging drop format. The final crystallisation conditions were 2.6–2.8 M ammonium sulphate, 0.1 M Hepes pH 7.0.

### Data collection, structure solution and refinement

All computations were carried out using programs from the CCP4 suite v. 7.0^47^. For the MGGMM-*Ypen*Man26A complex, data were collected at the Diamond Light Source beamline I04 to 1.36 Å resolution and processed using *xia2*^48^. The structure was solved using *MOLREP*^49^ with *Pans*Man26A (PDB entry: 3zm8; Couturier *et al*.^24^; sequence identity: 47.7%) as template. The structure was refined using REFMAC5^50^ iterated with manual model building/correction in Coot^51^. The final model was validated using Molprobity^52^ as part of the Phenix package^53^. Data-processing and refinement statistics are given in Table 1. Structure figures were prepared using CCP4*mg*^54^ or PyMOL v 1.7.20 (DeLano Scientific LLC, San Carlos, CA). The sequence alignments were created with MUSCLE^55^ and ESPript^56^.

### Homology modelling

The homology model of *Wsp*.Man26A was generated using HHPred-Homology server (https://toolkit.tuebingen.mpg.de/#/tools/hhpred)^57^ with *Pans*Man26A as template, (PDB ID: 3ZM8^24^, 54% sequence identity). Model quality was evaluated using the Ramachandran analysis in MolProbity (http://molprobity.biochem.duke.edu/)^52^. The model of *Wsp*.Man26A had 96.4% (430/437) of all residues in allowed regions. The model was only used to visualise the mutated amino acids in *Ypen*Man26A, which were inspired by *Wsp*.Man26A (Fig. 3).

### Thermal stability

The thermal stability at pH 5.0 was investigated with Differential Scanning Calorimetry (DSC) following an established protocol^26^. The Thermal midpoint (*Tm*) was determined as the top of the protein denaturation peak, with an accuracy of +/−1 °C.

### Initial rates and analysis of product profiles by DASH

The initial rates on locust bean gum and guar gum by the endomannanases were determined with 2.5 mg/ml substrate in 50 mM sodium acetate pH 5.0 at 37 °C. The hydrolytic activity was determined after 15 min in a 200 µl hydrolysis volume. Released reducing sugars were measured with the 4-hydroxybenzoic acid hydrazide (PAHBAH) method described by Lever^58^, with mannose as standard. All hydrolysis assays were carried out at 7 different endomannanase doses as described elsewhere^26^. Initial rates were calculated in the initial linear range of the hydrolysis. Guar gum hydrolysis product profiles at high conversion (26–36%) were analysed by DASH after inactivation by heating at 95 °C for 15 min. APTS (9-aminopyrene-1,4,6-trisulfonate) labelling and analysis of the labelled saccharides were carried out as described elsewhere^26,39^.

### Kinetics with locust bean gum and guar gum

The kinetic constants for locust bean gum and guar gum hydrolysis were determined by assaying the initial endomannanase rates at different substrate concentrations (10 to 0.1 mg/ml) using the PAHBAH assay as described above. The enzyme concentrations used for the locust bean gum hydrolysis were 4 nM *Ypen*Man26A wild-type, 4 nM *Wsp*.Man26A, 4 nM *Ypen*Man26A D37T, and 18 nM *Ypen*Man26A W110H and for the guar gum hydrolysis were 4 nM *Ypen*Man26A, 10 nM *Wsp*.Man26A, 6 nM *Ypen*Man26A D37T, and 44 nM *Ypen*Man26A W110H. The initial hydrolysis rate, *V*_i_, was plotted as a function of the substrate concentration, [S]. Non-linear regression using the Michaelis-Menten equation was used to determine the values for *k*_cat_, *K*_M_ and *k*_cat_/*K*_M_.

### Kinetics with MGGMM

*k*_cat_/*K*_M_ was determined by following MGGMM depletion over time at low substrate concentration (0.1 mM), pH 5 and 37 °C, with an online, direct injection, mass spectrometry based assay. Duplicate samples were analysed using a HPLC-MS system with a Dionex Ultimate 3000RS HPLC connected to an ESI-iontrap (AmaZon SL, Bruker Daltonics). The HPLC provided a constant flow of 0.1 ml/min of 50/50 vol-% acetonitrile and 0.1% formic acid. The electrospray was operated in positive ultrascan mode with Multiple Reaction Monitoring (MRM) using a target mass of *m/z* 800. MRM mode was chosen to selectively follow substrate depletion and an internal standard (XXXG). 100% reaction amplitude was used to ensure fragmentation of the precursor ion. The capillary voltage was set at 4.5 kV, end plate offset was 0.5 kV, nebulizer pressure 3.0 bar, dry gas flow 12.0 l/min, and dry gas temperature was set to 280 °C. Buffer concentration, 1 mM sodium acetate pH 5, was set as low as possible to minimize ion suppression without compromising pH in the reaction. The total reaction volume was 500 µl and the sample was incubated directly in an HPLC-vial in the HPLC-autosampler. The reaction was started by adding enzyme in 2 nM and 6 nM for the wild-type *Ypen*Man26A and the D37T variant respectively. Two min after enzyme addition, the first sample was taken. Thereafter, sampling was performed every 5.4 min (including sampling procedure), when the autosampler injected 4 µL sample directly into the flow leading to the MS. The enzyme reaction was immediately quenched when entering the flow path because the mobile phase was pH 2.7 and detection occurred approx. 0.5 min after injection. Total acquisition time was set to 4 min. The enzyme reactions were followed for a maximum time period of 50 min, but only data describing the initial phase of the reaction (less than 25% conversion of substrate) were used for estimating *k*_cat_/*K*_M_. Details on extracted ion chromatograms used for quantification of MGGMM and XXXG can be seen in Fig. S4. Data were analysed and quantified using Compass DataAnalysis 4.2 and Compass QuantAnalysis 2.2 provided by Bruker Daltonics. Ln (*S*_0_/*S*_*t*_) was plotted as a function of time (*t*) (Fig. S5) and *k*_cat_/*K*_M_ was calculated as described by Matsui *et al*.^40^; k = Ln (S_0_/S_t_), where *k* = ((*k*_cat_/*K*_M_)·[enzyme])·*t*, *S*_0_ = substrate concentration at time zero and *S*_*t*_ = substrate concentration at time *t*.

### Productive M5 binding modes

The hydrolytic cleavage pattern of M5 was determined for the *Ypen*Man26A wild-type and the W110H variant, by the previously established ^18^O-water product labelling methodology^24,42^. First, M5 hydrolysis products were analysed and quantified by high performance anion exchange chromatography with pulsed amperometric detection (HPAEC-PAD) using a Dionex ICS-5000 with a Carbo-Pac PA-200 column and guard column. For this, double incubations of 1 mM M5 and 50 nM wild-type enzyme or 200 nM W110H mutant in 1.5 mM sodium acetate buffer, pH 5 were stopped by boiling at timed intervals (30 min to 3 h). Data after 30 min incubation for *Ypen*Man26A or 2 h for the W110H mutant (approximately 30% hydrolysis) were used. The quantification allowed distinguishing between productive M5 binding modes that generated M4 and M1 versus those that generate M3 and M2. However, HPAEC alone cannot distinguish between the two possible binding modes generating M4 and M1 (i.e. binding either from subsite −4 to +1 or from −1 to +4), neither the two binding modes that generate M3 and M2 (i.e. binding from subsite −3 to +2 or −2 to +3). Therefore, incubations as above were also set up at 8 °C using 97% H_2_^18^O as stock solvent reaching 92% ^18^O-water in the reactions. Duplicate reactions were stopped after 30 min (for wild-type) and 2 h (for W110H) by directly spotting 0.5 μl samples with 0.5 ml matrix (10 mg/ml 2,5-dihydroxybenzoic acid) on a stainless-steel plate, followed by immediate drying with warm air. Spectra were then obtained by MALDI-TOF MS and used to calculate the ^18^O over ^16^O product ratios using the monoisotopic peak areas as previously described^24,42^. Since M5 hydrolysis in ^18^O-water generates products where the newly formed reducing end becomes ^18^O-labelled (and other chain ends do not), the ^18^O over ^16^O product ratios can be used to calculate the relative frequency of the productive binding modes mentioned above (i.e. M5 binding from subsite −4 to +1 versus subsite −1 to +4 or binding from subsite −3 to +2 versus subsite −2 to +3)^24,42^. The procedure involves two calculated corrections for the product ratio determination, one for the (M + 2) natural isotope peak of the light (^16^O) species which overlaps with the heavy (^18^O) peak and a second for the presence of 8% ordinary H_2_^16^O in the hydrolysis reaction.

## Supplementary information

Supplementary material

## Data Availability

All data generated or analyzed during this study are included in this article and its Supplementary Information file.
