# Supplementary material for: Crystal structure and substrate interactions of an unusual fungal non-CBM carrying GH26 endo-β-mannanase from Yunnania penicillata
Source: Sci Rep. 2019 Feb 19;9:2266. doi: 10.1038/s41598-019-38602-x (PMC6381184; doi:10.1038/s41598-019-38602-x)
Supplement: Supplementary file 1 — Supplementary material [file 41598_2019_38602_MOESM1_ESM.pdf]

Supplementary material

Crystal structure and substrate interactions of an unusual fungal non-CBM carrying GH26 endo-β-mannanase from *Yunnania penicillata*

Pernille von Freiesleben, Olga V. Moroz, Elena Blagova, Mathias Wiemann, Nikolaj Spodsberg, Jane W. Agger, Gideon J. Davies, Keith S. Wilson, Henrik Stålbrand, Anne S. Meyer and Kristian B. R. M. Krogh

Lane	Sample
1	LMW – 10µl
2	*Ypen*Man26A E165Q – 5µl
3	*Ypen*Man26A E165Q + EndoH– 5µl
4	LMW – 10µl
5	LMW – 10µl
6	*Ypen*Man26A – 5µl
7	*Ypen*Man26A + EndoH– 5µl
8	LMW – 10µl

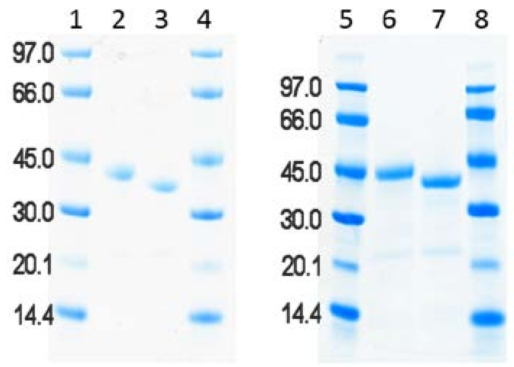

Figure S1. SDS-PAGE before and after EndoH treatment of the active and inactive *Ypen*Man26A. The protein concentration in the samples were 0.5 mg/ml. Prior to gel loading, samples were diluted 1:1 with loading mix. Loading mix was prepared as a 9:1 mix of Novex ® Tris-Glycine SDS Sample Buffer (2X) (Life Technologies) and Nupage ® Sample Reducing Agent (10X) (Life Technologies). Values 14.4 - 97.0 indicate molecular weights in kDa of bands in the LMW standard (lanes 1, 4, 5, 8). Please consult the manuscript for enzyme abbreviations.

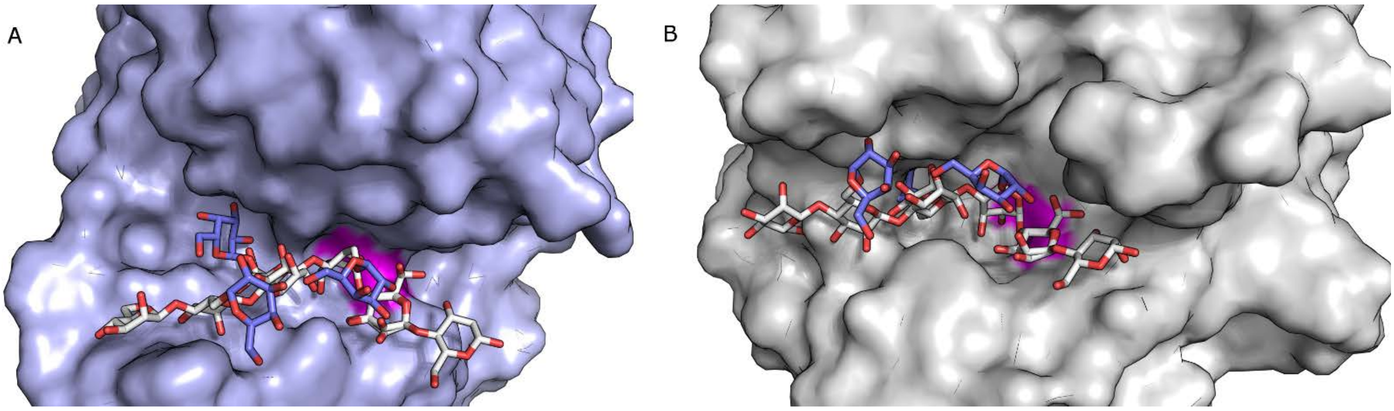

Figure S2. Surface views of the crystal structures of (A) *Ypen*Man26A from *Yunnania penicillata* and (B) *Cjap*Man26C (2VX6) from *Cellvibrio japonicus* showing the architecture of their active site cleft. A superimposition of the two structures allowed visualization of ligands from both crystal structures in each structure: α-6^2-6^1-di-galactosyl-mannotriose (MGG) binding from the -4 to -2 subsites in *Ypen*Man26A and α-6^3-galactosyl-mannotetraose (MGMM) binding from the -2 to +2 subsites in *Cjap*Man26C. Mannose units are coloured white and the galactose substitutions are coloured blue. Catalytic residues are shown in magenta.

Lane	Sample
1	LMW - 10µl
2	*Ypen*Man26A D37T – 10µl
3	*Ypen*Man26A W110H – 10µl

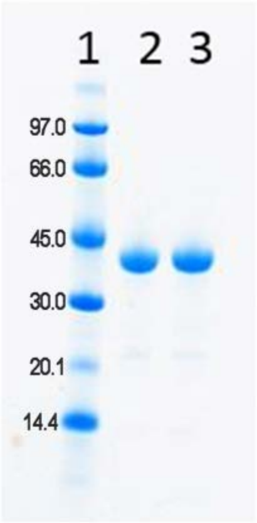

Figure S3. SDS-PAGE of purified *Ypen*Man26A mutants. Prior to gel loading, samples were diluted 1:1 with loading mix. Loading mix was prepared as a 9:1 mix of Novex ® Tris-Glycine SDS Sample Buffer (2X) (Life Technologies) and Nupage ® Sample Reducing Agent (10X) (Life Technologies). Values 14.4 - 97.0 indicate molecular weights in kDa of bands in the LMW standard (lane 1). Please consult the manuscript for enzyme abbreviations. The purification of *Wsp.*Man26A is described by von Freiesleben et al 2018.

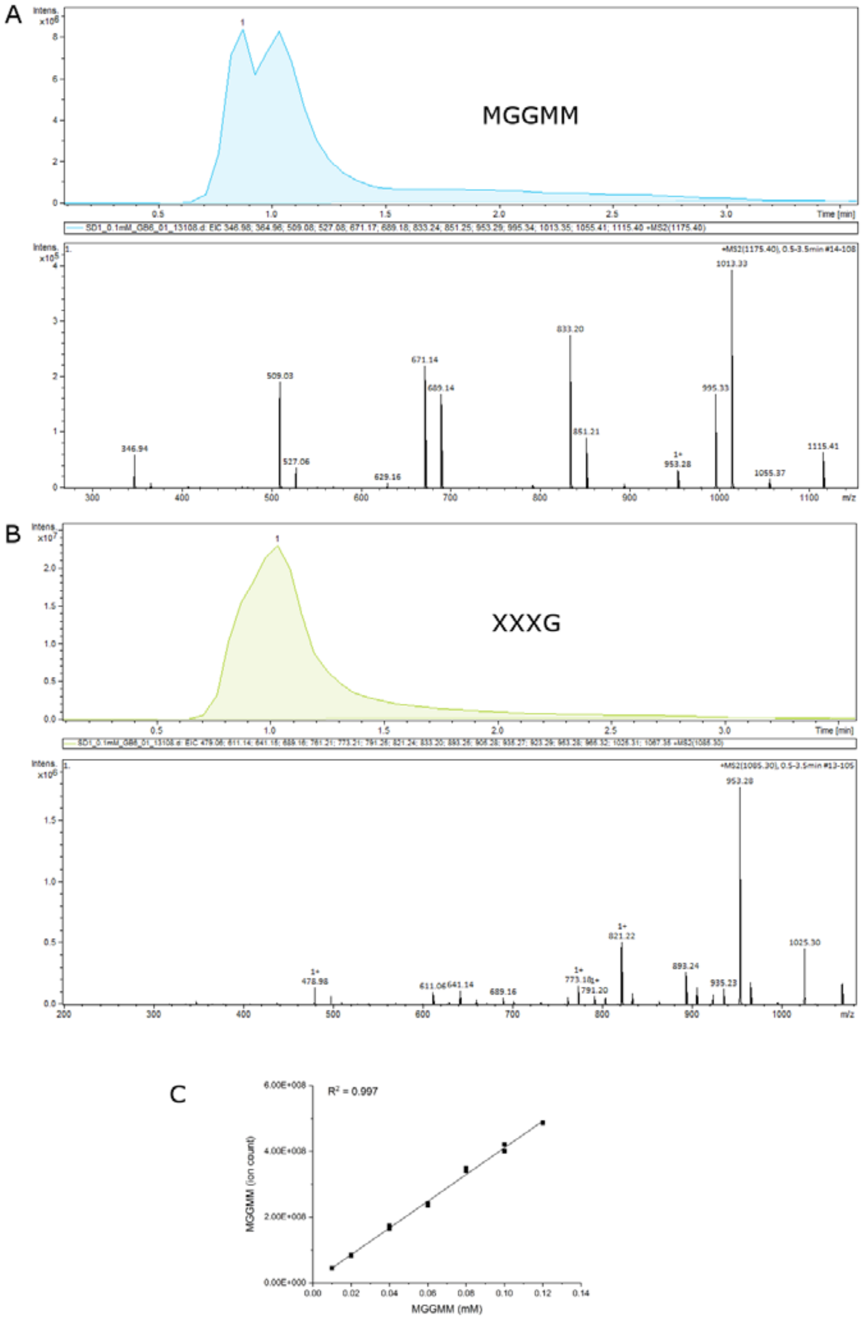

Figure S4. The observed precursor ion of both α-6^4-6^3-di-galactosyl-mannopentaose (MGGMM) and the internal standard (α-6^2-6^3-6^4-tri-xylosyl-glucotetraose) XXXG was the single charged sodium adduct $[M+Na]^+$of *m/z* 1175.4 and 1085.3 respectively. Data were collected with a window width of 0.5 amu. XXXG was added to the enzyme reaction as an internal standard to verify consistent signal response. XXXG was chosen because the mass and the branched structure are similar to MGGMM and because the enzymes in question did not have any activity towards it. The extracted ion chromatogram (EIC) and fragmentation pattern of $[M+Na]^+$ is shown for (**A**) 0.1 mM MGGMM (fragmentation ions used for quantification after MS/MS was *m/z* 346.9800; 364.9589; 509.0800; 527.0800; 671.1700; 689.1800; 833.2400; 851.2500; 953.2900; 995.3400; 1013.3500; 1055.4100; 1115.4000) and (**B**) 0.1 mM XXXG (fragmentation ions used for quantification after MS/MS was *m/z* 479.0600; 611.1400; 641.1500; 689.1600; 761.2052; 773.2100; 791.2500; 821.2400; 833.2031; 893.2500; 905.2779; 935.2700; 923.2869; 953.2800; 965.3159; 1025.3100). Data was obtained with an online, direct injection mass spectrometry based assay in positive ultrascan mode with Multiple Reaction Monitoring (MRM) following only MGGMM and XXXG. Peak integration was performed manually from 0.5 – 3.5 min. (**C**) A standard curve with seven calibration levels for MGGMM (0.01-0.12 mM) using XXXG as internal standard was used for quantification. Values are shown from two individual replicates, with a linear fit.

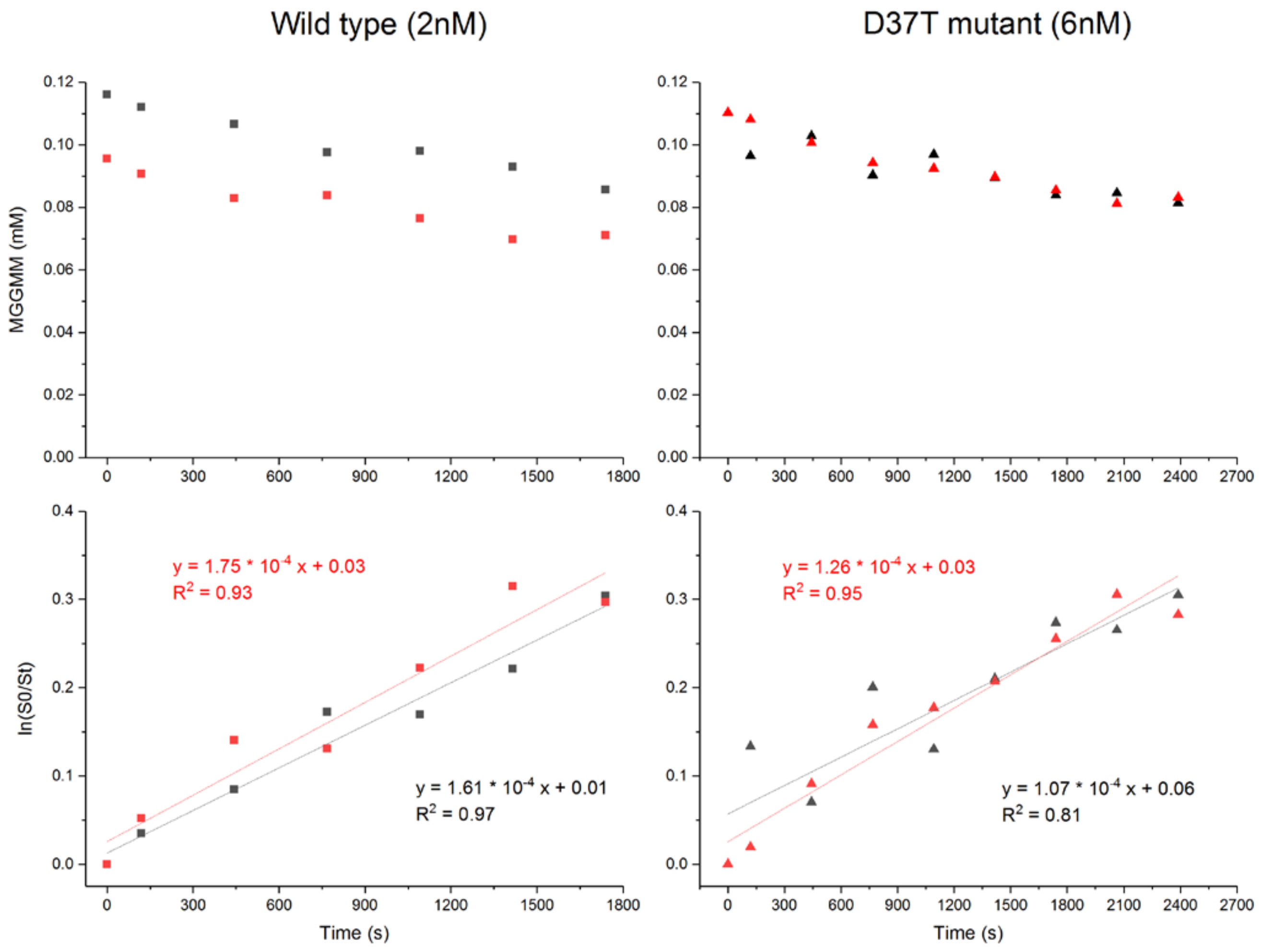

Figure S5. *Ypen*Man26A wild-type (**left**) and D37T mutant (**right**) catalysed MGGMM depletion. Data are plotted as **(top)** Substrate (*S*) as a function of time (*t*) to show MGGMM depletion and (**bottom**) as Ln(S_0/S_t) as a function of *t* to illustrate the Matsui equation. Values are shown from two individual replicates (black and red), with a linear fit for each replicate in the Matsui graph (bottom). k_{cat}/K_M can be calculated according to the Matsui equation; k = Ln(S_0/S_t) where $k = ((k_{cat}/K_M)$*[enzyme])*t, S_0 = substrate concentration at time zero and S_t = substrate concentration at time t (Matsui et al. 1991). Enzyme catalysed MGGMM depletion is not necessarily linear and it is not a prerequisite to use only the initial hydrolysis rate for the Matsui method.

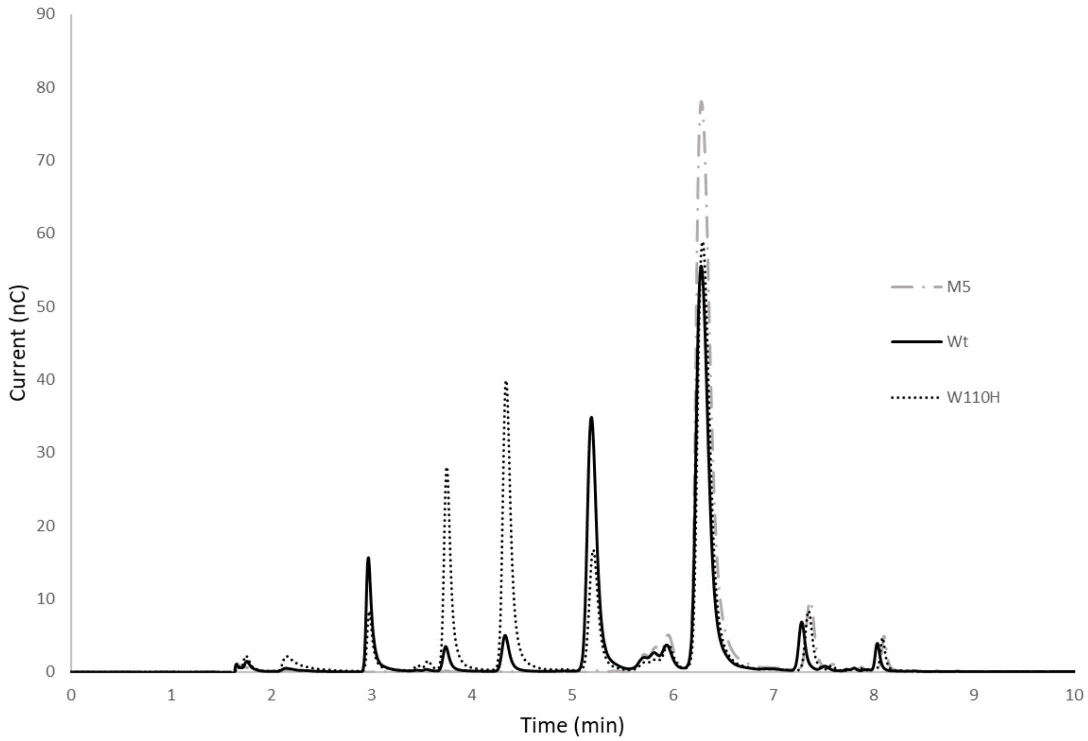

Figure S6. Representative HPAEC chromatogram of M5 hydrolysis by *Ypen*Man26 wild-type and W110H mutant. The solid line shows M5 hydrolysis by *Ypen*Man22. The dotted line shows M5 hydrolysis by the W110H mutant. The dotted and dashed line is a control sample containing the M5 substrate without enzyme and incubated under the same conditions.
